# Macrophage Polarization in Cerebral Aneurysm: Perspectives and Potential Targets

**DOI:** 10.1155/2017/8160589

**Published:** 2017-12-27

**Authors:** Lingmin Shao, Xingping Qin, Jia Liu, Zhihong Jian, Xiaoxing Xiong, Renzhong Liu

**Affiliations:** Department of Neurosurgery, Renmin Hospital of Wuhan University, Wuhan, China

## Abstract

Cerebral aneurysms (CAs) have become a health burden not only because their rupture is life threatening, but for a series of devastating complications left in survivors. It is well accepted that sustained chronic inflammation plays a crucial role in the pathology of cerebral aneurysms. In particular, macrophages have been identified as critical effector cells orchestrating inflammation in CAs. In recent years, dysregulated M1/M2 polarization has been proposed to participate in the progression of CAs. Although the pathological mechanisms of M1/M2 imbalance in CAs remain largely unknown, recent advances have been made in the understanding of the molecular basis and other immune cells involving in this sophisticated network. We provide a concise overview of the mechanisms associated with macrophage plasticity and the emerging molecular targets.

## 1. Introduction

Cerebral aneurysms (CAs) are a major cause of subarachnoid hemorrhage (SAH) [1]. Up to 50% of SAH patients die within the first 30 days after aneurysm rupture, and 30–50% survivors suffer from moderate-to-severe disabilities [2]. Clarification of mechanisms underlying the pathogenesis of CA is fundamental for developing effective therapies. In recent years, it is well recognized that inflammation plays an etiological role in the formation and rupture of CAs [3, 4], though several other factors mainly hemodynamic, genetic, environmental, and hormonal have been identified [5–8]. In particular, macrophages have been confirmed as critical effector cells in the progression of CAs [9]. In animal models, both macrophage depletion and inhibition of monocyte chemotactic protein-1 (MCP-1), a key chemoattractant of macrophages, are associated with a reduced incidence of CAs [10]. Macrophages are not homogeneous, and they are generally categorized into two subsets known as classically activated macrophages (M1-like) and alternatively activated macrophages (M2-like), respectively [11]. In general, M1 cells exhibit a proinflammatory effect while M2 cells facilitate resolution of inflammation and promote tissue repair. In response to various environmental cues (e.g., microbial products, damaged cells, and activated lymphocytes), macrophages can acquire distinct functional phenotypes via undergoing different phenotypic polarization, which are finely regulated processes [12, 13]. Their imbalances have been thought to be associated with various diseases [14]. Hasan et al. found that M1 and M2 cells were present in equal proportions in unruptured aneurysms; however, a marked predominance of M1 over M2 cells was documented in ruptured aneurysms [15]. Therapies targeting macrophage activation or preventing the M1/M2 imbalance may potentially halt aneurysm formation and rupture. In this review, we will focus on the factors that influence macrophage polarization in CAs. We will also discuss potential targets for CA therapies.

## 2. Molecular Mechanisms of Macrophage Polarization in Cerebral Aneurysm

**Figure 1:**
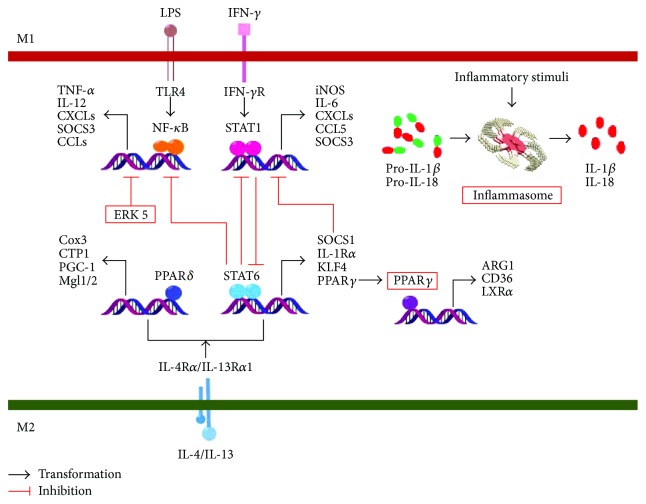
Mechanisms of macrophage polarization. Activation of IRF/STAT signaling pathways by IFN and TLR signaling skews macrophage function toward the M1 phenotype (via STAT1), while activation of IRF/STAT (via STAT6) signaling pathways by IL-4 and IL-13 skews macrophage function toward the M2 phenotype. PPAR*γ* and ERK 5 participate in the promotion of M2 macrophage in cerebral aneurysms. NLRP3 inflammasome may contribute to M1 polarization.

Extensive research efforts have been made in defining the molecular networks underlying macrophage polarization. As shown in Figure 1, IRF/STAT (interferon-regulatory factor/signal transducer and activator of transcription) signaling is a central pathway in modulating macrophage M1-M2 polarization. A detailed description of these processes is provided in the excellent recent reviews on this subject [12, 16]. Here, we focus on the molecular mechanisms of macrophage polarization in cerebral aneurysm.

Toll-like receptor signaling, particularly the activation of TLR4 (Toll-like receptor 4), drives macrophages to a preferential M1 phenotype in cerebral aneurysms [17, 18]. The signaling pathway through the Myd88 (myeloid differentiation primary response gene-88) adaptor results in the activation of IKK*β* (inhibitor kappa B kinase *β*). In addition, the activation of IKK*β* leads to the phosphorylation and degradation of I*κ*B (inhibitor kappa B), which permits the translocation of free NF-*κ*B (nuclear factor kappa-light-chain-enhancer of activated B cells) to the nucleus. As a key transcription factor related to macrophage M1 polarization, NF-*κ*B activates the expression of a large number of inflammatory genes, resulting in tissue damage [19]. On the other hand, M2 phenotype is promoted by several transcription factors. For example, a recent study has shown that the activation of PPAR*γ* (peroxisome proliferator-activated receptor γ) by pioglitazone promoted M2 activation to protect mice from CAs [20]. Besides, it has been reported that ERK5 (extracellular signal-regulated kinase 5) activation reduced the M1/M2 ratio by inhibiting the NF-*κ*B pathway in CAs [21]. Although these promising results expand our knowledge of macrophage polarization in CAs, the molecular mechanisms that govern the phenotype switch of macrophages remain largely unknown. Recently, NLRP3 (nucleotide-binding domain and leucine-rich-repeat-containing protein 3) inflammasome has been detected in T cells and macrophages in the tissue of human CAs [22]; however, it remains unclear how NLRP3 inflammasome further regulates macrophage polarization. In addition, the role of miRNA (microRNA) in the development of cerebral aneurysm has been of particular interest. Several miRNAs (e.g., miRNA-133, miRNA-140-3p, and miRNA-145-5p) involved in the differentiation of macrophages have been identified in CAs, but their targets need further investigation [23].

## 3. Classically Activated Macrophages in Cerebral Aneurysm

Cerebral aneurysms are characterized by disruption of the internal elastic lamina (IEL), phenotypic modulation of smooth muscle cells (SMCs), apoptosis of mural cells, and extracellular matrix (ECM) degradation, which are considered as the hallmarks of CA [24]. Mechanistic links between chronic inflammatory response and these features have been provided by repeated animal studies [25, 26]. Cerebral aneurysm development is characterized by increasing polarization towards the M1 macrophage phenotype. Nowicki and coworkers have reported that the M1 to M2 macrophage phenotype ratio increased during the 2-week period as aneurysms developed in mice [27]. Inflammatory cytokines derived from M1 cells initiate the pathological changes of aneurysmal walls, especially mediated by tumor necrosis factor *α* (TNF-*α*) [28, 29] and interleukin-1*β* (IL-1*β*) [30]. For example, TNF-*α*, an essential cytokine in the pathogenesis of CAs, initiates SMC phenotypic modulation that is an alteration from contractile to a proinflammatory and matrix remodeling phenotype [31]. In addition, IL-1*β* inhibits ECM biosynthesis in SMCs, thereby exacerbating degeneration of CA walls [32]. Sustained inflammatory response may eventually trigger apoptosis of mural cells, which ultimately leads to aneurysm rupture. On the other hand, the irritated SMCs further propagate the inflammatory cascades by secreting cytokines [31], which drive macrophages to M1 polarization. The interaction between M1 macrophages and SMCs may exacerbate the progression of CA through a positive feedback loop.

## 4. Inducing Alternative Activation of Macrophages Relieves Inflammation in CAs

Sustained chronic inflammation may result from dysregulated macrophage polarization. Macrophages can be driven to M2 phenotype not only by canonical M2 stimuli (e.g., IL-4, IL-13, and IL-10) but also by several transcription factors, including PPAR*γ* and Kruppel-like factor 4 (KLF-4) [13]. PPAR*γ* was identified as a critical factor in modulating macrophage M2 polarization induced by IL-4 or IL-13 [33, 34]. Recent study indicated that a PPAR*γ* agonist, pioglitazone, exhibited a protective effect on preventing CA rupture in mice [35]. Moreover, Shimada et al. reported that decreased infiltration of M1 macrophage into the CAs and the macrophage M1/M2 ratio were documented following pioglitazone treatment. Interestingly, the beneficial effect of pioglitazone treatment was abolished in macrophage-specific PPAR*γ* knockout mice. The authors concluded that activation of PPAR*γ* in macrophages may act against CA rupture through reducing macrophage-related cytokines, including IL-1, IL-6, and MCP-1 [20]. Their study sheds light on noninvasive treatment of CAs by inducing inflammation regression, such as promoting M2 shift. However, the underlying mechanisms governing these processes remain to be elucidated.

## 5. Regulation of Macrophage Plasticity by Other Immune Cells

**Figure 2:**
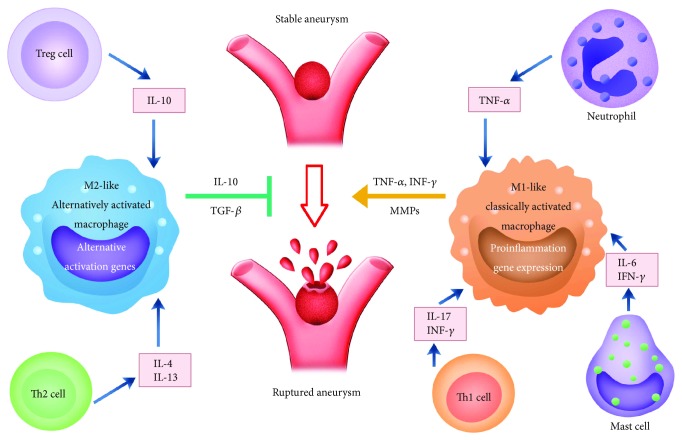
Summary of mediators and immune cells involved in M1/M2 polarization. The proinflammatory cytokines released by neutrophil, Th1 cells, and mast cells contribute to the maintenance of classically activated macrophage. Polarized M1 cells increase inflammation gene expression, promoting the progression of cerebral aneurysm to rupture. Conversely, alternatively activated macrophages may halt aneurysm rupture by facilitating inflammation regression. MMP indicates matrix metalloproteinase.

Besides macrophages, the representation of several other immune cell populations, such as neutrophils, natural killer (NK) cells, mast cells, and lymphocytes, is altered in CA walls [36]. As the initial responder to cellular stress, macrophages can contribute to the further recruitment and activation of adaptive immune cells. These immune populations elicit their effects on the potentiation or repression of inflammation by altering the activation state of macrophages, suggesting a highly complex regulation of the inflammatory processes in vascular wall (Figure 2). Over the last decade, orchestration of inflammation by these immune cells in CAs has been extensively investigated. For example, it has been reported that neutrophil blockade using anti-CXCL1 (C-X-C motif ligand 1) antibody attenuated polarization towards the M1 phenotype during the 2 weeks postaneurysm induction in mice, suggesting that CXCL1-dependent neutrophil inflammation may have an important role in macrophage polarization to M1 phenotype in the development of CAs [27].

Although best known for the contribution of mast cells in microbial defense and allergy, previous study has found that mast cells were invariably present in CA walls and were more pronounced in ruptured than in unruptured human CAs [15]. Reduced infiltration and activation of mast cells effectively attenuate destruction in aneurysmal walls, suggesting their roles in CA development [37]. Degranulation of mast cells led to increased expression of MMP-2 (matrix metalloproteinase-2) and MMP-9 and induced nitric oxide synthase, which result in damage to the vascular wall [38]. Moreover, they release cytokines, including TNF-*α*, IL-1*β*, and MCP-1, which potently activate M1 macrophages. By using mast cell degranulation inhibitors, decreased macrophage infiltration was evident in a rat model [37]. However, the biological mechanisms underlying interaction between M1 macrophages and mast cells remain unclear. Further studies are needed to determine the potential role of mast cells in macrophage polarization and the pathology of CAs.

Studies of specimens of human CAs have shown that both T and B lymphocytes robustly infiltrate the vessel wall, especially around the site of CA rupture; presumably, they are involved in the progression of CAs [36]. Nonetheless, the role of lymphocytes in the pathogenesis of CAs is controversial. Sawyer et al. found that CA formation and rupture in lymphocyte-deficient mice were significantly less prevalent than that in wild-type group, though they were equally subjected to a robust CA induction protocol [39]. Conversely, a recent study indicated that deficiency of T cells in rats failed to affect CA progression, degenerative changes of arterial walls, and macrophage infiltration in lesions [40]. As T lymphocytes can differentiate into distinct subsets following the local stimuli within the CA walls, it is tempting to speculate that a certain subset of T cells may contribute to the pathogenesis of CAs. In clinical, patients with CAs exhibited a CD4^+^ T cell skewing in their peripheral blood, with more Th17 (T helper cell 17) and fewer Th2 cells. In line with these findings, IL-17 level was elevated while IL-10 was decreased. Although the representation of Th1 and Treg cells (regulatory T cells) in CA patients was not distinguished from that of healthy controls, altered cytokine profiles were detected. In patients suffered from CAs, the Th1 cytokines (IFN-*γ*, TNF-*α*) were increased whereas the production of IL-10 was declined significantly [41]. The imbalance of CD4^+^ T cell was likely to facilitate inflammation in CAs. Their findings do not fully describe the range of functions that activated macrophages exert, but specialized T cells (Th1, Th2, Th17, and Treg cells) presumably participate in macrophage polarized activation [42, 43]. Considering their crucial roles in adaptive immune response, the effect of specific T subsets on macrophage polarization remains to be revealed.

## 6. Current Antiinflammatory Therapeutic Strategies and Future Directions

Since chronic inflammation is a key etiologic factor in CA formation and rupture, therapeutic attempts to interfere with inflammatory response have potential importance. Several clinical agents have been investigated with varied success, perhaps the most promising one being aspirin [44]. Both direct macrophage imaging and histological examination have confirmed that aspirin ameliorates the inflammation of CA walls in human [45, 46]. Growing evidences indicate that administration of aspirin is associated with the reduced risk of CA rupture in humans [47, 48]. A detailed discussion of this subject can be found in recent reviews [49, 50].

Given the critical role of macrophages in the etiology of cerebral aneurysm to rupture, macrophage-mediated therapies, by directly effecting on macrophages or indirectly targeting other immune cells that regulate M1/M2 polarization, are likely to represent novel strategies for CA treatment [16]. As mentioned above, PPAR*γ* was identified as a key factor inducing alternative M2 phenotype. In human atherosclerotic lesion, PPAR*γ* activation primes monocyte toward an alternative M2 phenotype [51]. In parallel, reduced infiltration of M1 macrophage and the M1/M2 ratio are observed following pioglitazone in mouse model, raising the possibility that inflammatory cell PPAR*γ* is emerging as a potential target for preventing CA rupture. Recently, NLRP3 inflammasome, a multiprotein complex initiating the maturation of pro-IL-1*β* and pro-IL-18, is detected in T cells and macrophages within the wall of human CAs [22]. Activation of NLRP3 inflammasome results in IL-1*β* and IL-18 production, which potently induce M1 polarization. It has been confirmed that Nlrp3-knockout mice show decreased M1 but increased M2 gene expression in adipose tissue macrophages [52]. These studies implicate that genetic elimination of the components of NLRP3 inflammasome may dampen the inflammatory response mediated by M1 macrophage. In contrast to recruitment of monocytes to arterial walls, the process of macrophage emigration from CAs may be impaired. In murine models of atherosclerosis, Netrin-1 was found to block macrophage movement by inhibiting actin reorganization, making cells refractory to emigration from plaques [53]. The mechanisms preventing macrophage egress from CAs warrant further exploration. Finally, with our refined recognition of the complex interactions between macrophages and other immune cells in CA wall, we are likely to enter a new era in which immune modulation can be proposed as a therapeutic strategy against cerebral aneurysm.

## 7. Concluding Remarks

In recent years, progress has been made in our understanding of dysregulated macrophage polarization in CAs; however, detailed processes remain fragmentary. It is likely that in the next few years, ongoing work in this field will continue. Future studies to delineate the mechanisms involving macrophage plasticity in the environment of aneurysmal walls will enable new strategies for attacking CAs.

## Abbreviations

CA: Cerebral aneurysm
SAH: Subarachnoid hemorrhage
MCP-1: Monocyte chemotactic protein-1
IRF: Interferon-regulatory factor
STAT: Signal transducer and activator of transcription
TLR4: Toll-like receptor 4
Myd88: Myeloid differentiation primary response gene-88
I*κ*B: Inhibitor kappa B
IKK*β*: Inhibitor kappa B kinase beta
NF-*κ*B: Nuclear factor kappa-light-chain-enhancer of activated B cells
PPAR: Peroxisome proliferator-activated receptor
ERK: Extracellular signal-regulated kinase
NLRP3: Nucleotide-binding domain and leucine-rich-repeat-containing protein 3
miRNA: MicroRNA
IEL: Internal elastic lamina
SMC: Smooth muscle cell
ECM: Extracellular matrix
TNF-*α*: Tumor necrosis factor *α*
IL-1*β*: Interleukin-1*β*
KLF-4: Kruppel-like factor 4
NK: Natural killer
CXCL1: C-X-C motif ligand 1
MMP: Matrix metalloproteinase
Th17: T helper cell 17
Treg: Regulatory T cell
TGF-*β*: Transforming growth factor-*β*
IFN-*γ*: Interferon-*γ*
SOCS3: Suppressor of cytokine signaling 3
iNOS: Inducible nitric oxide synthase.
